# Fibre composition in sow diets influences bile acid profile in colostrum and in intestinal digesta of their new-born suckling piglets

**DOI:** 10.1017/jns.2025.10050

**Published:** 2025-11-04

**Authors:** Łukasz Marcin Grześkowiak, Ignacio Rodolfo Ipharraguerre, Gerald Rimbach, Wilfried Vahjen, Jürgen Zentek

**Affiliations:** 1 Institute of Animal Nutrition, Freie Universität Berlin, Berlin, Germany; 2 Institute of Human Nutrition and Food Science, University of Kiel, Kiel, Germany

**Keywords:** Bile acids, *Clostridioides difficile*, lignocelluloses, microbiota, offspring, piglet, sugar beet pulp, BA, bile acids, FXR, farnesoid X receptor, GLP1, glucagon-like-peptide-1, IgG, immunoglobulin G, LNC, lignocellulose, SCFA, short-chain fatty acids, TGR5, takeda G protein-coupled receptor 5, HPLC-QQQ-MS, high-performance liquid chromatography coupled to triple quadrupole mass spectrometry, PCNA, proliferating cell nuclear antigen, SBP, sugar beet pulp, TGF-β, transforming growth factor β, ZO-1, zonula occludens 1, LCA, lithocholic acid, DCA, deoxycholic acid, CDCA, chenodeoxycholic acid, HDCA, hyodeoxycholic acid, UDCA, ursodeoxycholic acid, CA, cholic acid, HCA, hyocholic acid, GCA, glycocholic acid, TDCA, taurodeoxycholic acid, TCA, taurocholic acid, TCDCA, taurochenodeoxycholic acid, THDCA, taurolithodeoxycholic acid, TUDCA, tauroursodeoxycholic acid, THCA, 3α,7α,12α-trihydroxycholestanoic acid, GCDCA, glycochenodeoxycholic acid, GHDCA, glycohyodeoxycholic acid, GHCA, glycohyocholic acid, OLCA, oxalolithocholic acid, TLCA, taurolithocholic acid

## Abstract

Dietary fibre can modify colostrum and milk composition in sows. Bile acids (BA) aid in fat digestion and lipid absorption and are important signalling molecules for the digestive tract. The aim of this study was to determine BA concentration in colostrum from sows fed two different sources of dietary fibre during gestation and lactation and from the intestinal digesta of their 4-6-days-old suckling offspring. Twenty sows were fed diets enriched with either 15% high-fermentable sugar beet pulp (SBP, *n* = 10) or 15% low-fermentable lignocellulose (LNC, *n* = 10). Sow colostrum, piglet gallbladder content, ileum and colon digesta were assessed for BA using high-performance liquid chromatography coupled to triple-quadrupole mass spectrometry. In colostrum, lithocholic acid and oxolithocholic acid were higher in sows fed SBP vs. LNC (*p* = 0.005 and *p* = 0.003, respectively), while 3α,7α,12α-trihydroxycholestanoic acid and glycohyodeoxycholic acid were higher in colostrum from sows fed LNC vs. SBP (*p* = 0.039, *p* = 0.002, respectively). In the piglet bile, cholic acid and taurodeoxycholic acid were higher in SBP vs. LNC group (*p* = 0.02, *p* = 0.001, respectively), while taurochenodeoxycholic acid was higher in LNC vs. SBP group (*p* = 0.035). In the piglet ileum digesta, lithocholic acid was higher in SBP vs. LNC (*p* = 0.015). In the piglet colon digesta, lithocholic acid and ursodeoxycholic acid were higher in SBP vs. LNC (*p* = 0.001 and *p* = 0.007, respectively). Addition of specific dietary fibres to sow diets differentially influences the BA in colostrum. Dietary fibres in sow diets can impact on the intestinal BA composition in piglets with a possible consequence on the digestive physiology and health in the offspring.

## Introduction

Diet has a profound and long-lasting impact on the host and intestinal microbiota. Dietary fibre is an essential component of pig diets. During fermentation, complex carbohydrates are broken down by the gut microbiota into simple sugars, which are then metabolised into short-chain fatty acids (SCFA). Specifically, fibre-rich diets have been shown to influence the gut physiology and health of sows and their offspring.^(1)^ Bile acids (BA) are synthesised in the liver and stored in the gallbladder, where they are released into the small intestine to aid in the emulsification and absorption of dietary lipids. Thereafter, they are reabsorbed and returned to the liver *via* the enterohepatic circulation. However, about 2–5% of the BA escape absorption in the small intestine and enter the hindgut, where they are metabolised by the gut microbiota.^(2)^ BA, besides their crucial role in fat digestion and lipid absorption, are important signalling molecules for the digestive function, cell proliferation, cancer promotion and the immune system.^(3,4)^ The link between BA and signalling through farnesoid X receptor (FXR) and takeda G protein-coupled receptor 5 (TGR5) is integral to the regulation of BA synthesis, enterohepatic circulation, and associated metabolic processes. The activation of these receptors by specific BA acts as a signalling mechanism to coordinate various aspects of metabolism such as glucose homeostasis but also to maintain BA homeostasis and promote gut barrier function.^(3,5)^

In conventional conditions, piglets are not fed fibre-rich diets and therefore, reports on the impact of fibre-rich diets on the BA composition in piglets are still scarce.^(6)^ The type of fibre can differentially influence the metabolic activity of the microbiota and therefore their potential to hydrolyse conjugated BA.^(7,8)^ The detergent action of conjugated BA can control microbial populations and infection development by directly affecting the bacterial growth and cell wall integrity. For instance, taurocholic acid, a primary BA, is a very effective germinant for *Clostridioides difficile* spores, while the deconjugated secondary BA lithocholic acid and deoxycholic acid can inhibit the growth and toxin production of *C. difficile* vegetative cells.^(9–11)^

New-born piglets derive vital nutrients and passive immunity from colostrum. Porcine colostrum is abundant in proteins, fats, lactose, and immunoglobulins. In contrast to mature milk, colostrum exhibits reduced levels of gross energy and contains lesser quantities of fat and lactose.^(12)^ Additionally, colostrum contains various biogenic amines that may function as signalling molecules for intestinal cells.^(13)^ The rapid digestion and absorption of nutrients from colostrum, coupled with the efficient passage of immunoglobulins through the gut barrier, contribute to their swift entry into the piglet’s bloodstream.^(14)^

The presence of bile acids in porcine colostrum has so far been reported in two recent publications.^(15,16)^ BA in colostrum could have several important biological functions in new-borns such as digestion of fat, maturation of the new-born’s digestive system, colonisation and assembly of the gut microbiota, early defence against potential pathogens, and stimulation of gut development. Thus, BA in colostrum might be considered also as a critical component for the development and protection of the new-born’s digestive and immune systems during the early stages of life. Our preliminary results on the BA in colostrum have been published in abstract form.^(17)^ It is known that dietary fibre can modulate gut microbiota-mediated BA metabolism, thereby altering the circulating BA pool.^(18)^ Since BA have been found in sow milk,^(15,16)^ shifts in gut BA profiles may translate into altered BA concentrations and composition in colostrum. Consequently, the dietary fibre intake of sows may influence neonatal exposure to BA during early life.

Hence, our hypothesis postulates that diets rich in either high- or low-fermentable fibre during gestation and lactation would have varying effects on the BA composition in the colostrum of sows and in the intestinal digesta of their suckling piglets. With this in mind, we quantified BA in sows’ colostrum and in the gallbladder bile, ileum and colon digesta of their four- to six-day-old piglets.

## Methods

### Animals and sampling

The institutional and national guidelines for the care and use of animals were followed and the study was approved by the State Office of Health and Social Affairs ‘Landesamt für Gesundheit und Soziales Berlin’ (LAGeSo Reg. G0112/19).

Twenty German Landrace sows were randomly allocated to two experimental feeding groups (3 multiparous and 7 primiparous per group). They were kept in groups of 10 animals/pen and housed individually in farrowing pens one-week ante-partum until weaning. The sows’ feed intake was restricted throughout gestation (2.5 kg/sow/day until 85 d, 3 kg/sow/day until 95 d and 3.5 kg/sow/day until farrowing) and *ad libitum* during the lactation period. Lactation diets were provided to sows from three days after the farrowing onwards. Water was available to the animals *ad libitum*. They were fed iso-energetic and iso-nitrogenous experimental gestation and lactation diets enriched with sugar beet pulp (SBP) (*n* = 10 sows) or lignocellulose (65% lignocellulose) (LNC) (*n* = 10 sows), as previously published^(7,13)^ (Table 1). The diets were formulated to meet or exceed the recommendations of gestating and lactating sows.^(19)^ As fibre content is essential for pig health,^(19)^ diets without dietary fibre supplementation could not be used as standard controls. The farrowing occurred naturally without artificial induction. Colostrum (10 mL) was collected manually (without oxytocin injection) within 10 h after beginning of the farrowing and once after the afterbirth was expelled. Colostrum aliquots were stored frozen at -20°C for further analysis. Briefly, colostrum from SBP-fed sows had higher crude protein and lower lactose levels compared to LNC-fed sows. Immunoglobulin (IgA, IgG, IgM) concentrations did not differ between the two groups, but total biogenic amines were higher in sows fed SBP compared to LNC. SBP-fed sows had numerically higher titres of IgG-anti-*Clostridioides*-*difficile*-toxin-A- and IgG-anti-*Clostridioides*-*difficile*-toxin-B-antibody titres in colostrum compared to LNC-fed sows.^(13)^

**Table 1.** Ingredients and chemical composition of the experimental diets

	Gestation diet		Lactation diet	
Ingredients (g/kg feed)	SBP	LNC	SBP	LNC
Sugar beet pulp^*^	150	25	150	25
Arbocel^†^	30	150	30	150
Barley	610	553	338	275
Soybean meal [49% CP]	77	111	171	204
Wheat	80	80	200	200
Premix^◊^	12	12	12	12
Calcium carbonate	7	11	9	10
Monocalcium phosphate	14	1	21	25
Lysine HCl	5	5	1	1
Soy oil	13	41	66	96
Salt	1	1	1	1
Threonine	1	1.3	1	1
Methionine	0.2	0.2	–	0.1
Calculated metabolisable energy (MJ/kg)	11.4	11.4	13.0	13.0
Analysed composition (g/kg fresh matter)				
Dry matter	899.5	902.4	918.0	917.5
Crude ash	46.0	43.7	65.0	61.2
Crude protein	138.9	138.8	165.6	157.2
Crude fat	30.4	42.6	80.5	99.1
Crude fibre	66.1	110.5	70.3	123.7
Neutral detergent fibre	163.4	225.9	180.1	230.0
Acid detergent fibre	85.9	143.9	79.3	125.4
Acid detergent lignin	9.6	33.5	10.9	34.2
Insoluble dietary fibre	188.4	245.1	187.6	244.2
Soluble dietary fibre	55.5	49.1	61.6	46.4
Total dietary fibre	243.8	294.2	249.2	290.6
Starch	400.0	355.8	345.7	289.1

SBP, sugar beet pulp-enriched diet; LNC, lignocellulose-enriched diet; CP, crude protein.

* SBP (containing approximately 78% of total and 41% of soluble NSP).^(43)^

† Arbocel® (containing approximately 65% of lignocellulose, J. Rettenmaier & Söhne GmbH & Co. KG, Rosenberg, Germany).

◊ Mineral and vitamin premix (Spezialfutter Neuruppin GmbH, Neuruppin, Germany), containing per kg DM: 130 g Na (as NaCl), 55 g Mg (as MgO), 210 mg retinol, 3 mg vitamin D_3_, 8 g DL-α-tocopherol, 300 mg menadione, 250 mg thiamine, 250 mg riboflavin, 400 mg vitamin B_6_, 2 mg vitamin B_12_, 2.5 g nicotinic acid, 100 mg folic acid, 25 mg biotin, 1 g pantothenate, 80 g choline chloride, 5 g Fe (as FeCO_3_), 1 g Cu (as CuSO_4_), 5 g Zn (as ZnO), 6 g Mn (as MnO), 45 mg I (as CaI_2_O_6_), 35 mg Se (as Na_2_SeO_3_).

New-born piglets were balanced for sex and weight (birth average body weights of further dissected piglets were 1,502 ± 82 g in SBP group and 1,633 ± 74 g in LNC group) and they were not provided with creep feed. Four to six days after farrowing, 20 piglets were euthanised. Each piglet belonged to different dam. In each feeding group, an equal number of female and male piglets were euthanised. Piglets from sows fed the diet supplemented with SBP exhibited an average body weight of 2,033 ± 115 g, while those from sows fed the diet supplemented with LNC had an average body weight of 2,112 ± 96 g (*p* = 0.603).^(13)^ The animals were sedated with 20 mg/kg BW of ketamine hydrochloride (Ursotamin®; Serumwerk Bernburg AG, Germany) and 2 mg/kg BW of azaperone (Stresnil®; Jansen-Cilag, Neuss, Germany). After sedation, the piglets were euthanized by intracardial injection of 10 mg/kg BW of tetracaine hydrochloride, mebezonium iodide and embutramide (T61®; Intervet, Unterschleißheim, Germany).^(20,21)^ After euthanasia, the gallbladder content, ileum and colon digesta were collected and stored frozen at -20°C until analysis.

### BA analysis

Extraction of BA was performed using water : acetonitrile (1:1) and chenodeoxycholic acid-d4 as the internal standard. Thereafter, the BA were quantified by high-performance liquid chromatography coupled to triple quadrupole mass spectrometry (HPLC-QQQ-MS). External standards in a range of concentrations between 0.001 to 5 µg/mL were as follows: cholic acid, deoxycholic acid, lithocholic acid, chenodeoxycholic acid, glycodesoxycholic acid, glycolic acid, taurodeoxycholic acid, taurocholic acid, hyodeoxycholic acid, ursodeoxycholic acid, and hyocholic acid. The separation process utilised a Kinetex XB-C18 100A column from Phenomenex, USA, with mobile phases consisting of 2 mM ammonium acetate in water and a mixture of acetonitrile and methanol (1:1). The separation was carried out at a constant flow rate of 1 mL/min and a temperature of 50 ºC. The HPLC system employed was an Agilent 1200 coupled with a Triple Quadrupole (QQQ) Agilent (G6410B). Data analysis was conducted utilizing Masshunter Qualitative Analysis (B.07.00), with quantification executed in the multiple reaction monitoring mode. This involved integrating ion areas according to standard curves using authentic standards, with chenodeoxycholic acid-d4 serving as the internal standard.^(22–24)^

### Validation and quality assurance

In accordance with the previously outlined operating parameters, we initiated the validation and quality assurance process by injecting external standards into the HPLC-QQQ-MS system. This step was used to establishing the fractionation profile prior to selection of species of interest using Multiple Reaction Monitoring (MRM) mode. In instances where uncertainty arose regarding the identified structure, the procedure was systematically repeated, and outcomes revised to ensure the accurate identification of both free and conjugated BA. Afterwards, linearity was established across the concentration range of 0.001 to 5 µg/mL for all external standards (R >0.99 for each). The limit of detection was greater than 1 ng/mL in every case and the limit of quantification exceeded 10 ng/mL. Recovery rates were determined to exceed 85 % for all tested standards, achieved through spiking samples with two levels of a multi-standard solution. To further verify the accuracy of the results, external multi-standards at various concentrations were injected into the system after every six samples.

### Statistical analyses

The experimental unit consisted of a single sow and a single piglet. The data for BA concentrations were normally distributed (Shapiro-Wilk test) and thus they were analysed using unpaired Student t-test. Significant differences were considered at *p* ≤ 0.05. Data were analysed using the Student-t-test in GraphPad Prism 9.0.2.

## Results

### BA composition in colostrum

**Figure 1:**
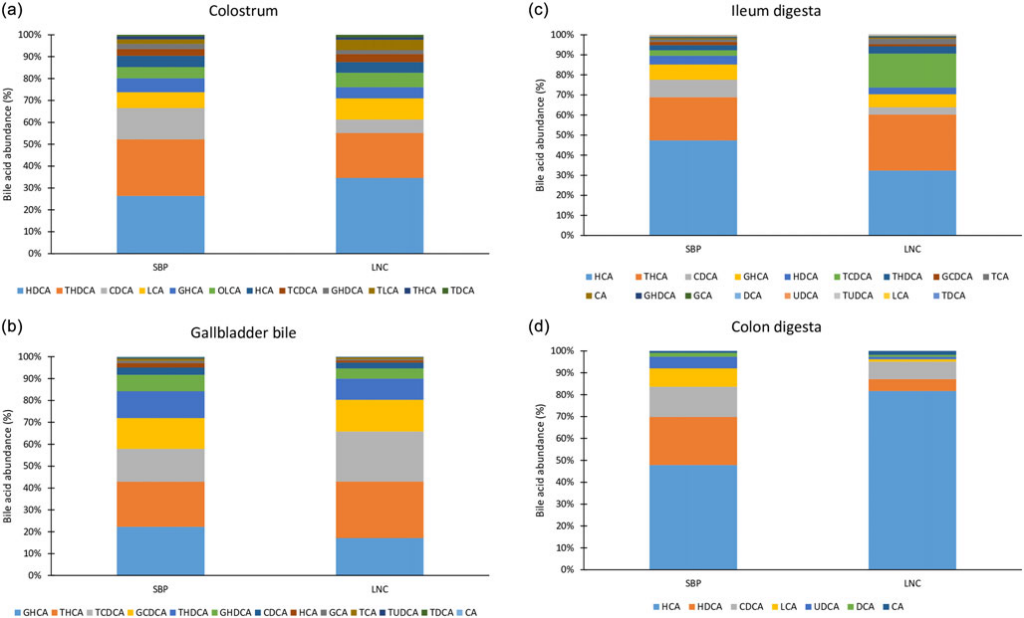
(a–d) Relative abundance (%) of the analysed bile acids in sow colostrum (a), gallbladder bile (b), ileum digesta (c) and colon digesta (d) from the piglets. SBP, sugar beet pulp; LNC, lignocellulose; LCA, lithocholic acid; DCA, deoxycholic acid; CDCA, chenodeoxycholic acid; HDCA, hyodeoxycholic acid; UDCA, ursodeoxycholic acid; CA, cholic acid; HCA, hyocholic acid; GCA, glycocholic acid; TDCA, taurodeoxycholic acid; TCA, taurocholic acid; TCDCA, taurochenodeoxycholic acid; THDCA, taurolithodeoxycholic acid; TUDCA, tauroursodeoxycholic acid; THCA, 3α,7α,12α-trihydroxycholestanoic acid; GCDCA, glycochenodeoxycholic acid; GHDCA, glycohyodeoxycholic acid; GHCA, glycohyocholic acid; OLCA, oxalolithocholic acid; TLCA, taurolithocholic acid.

In colostrum, among primary BA, 3α,7α,12α-trihydroxycholestanoic acid was significantly higher from sows fed LNC *vs*. SBP (*p* = 0.039). Among secondary BA, lithocholic acid and oxolithocholic acid were significantly higher in colostrum from sows fed SBP *vs*. LNC (*p* = 0.005 and *p* = 0.003, respectively), while glycohyodeoxycholic acid was significantly higher in colostrum from sows fed LNC *vs*. SBP (*p* = 0.002). The concentration of total, primary and secondary BA did not differ between the colostrum collected from sows fed SBP or LNC (Table 2). Taurodeoxycholic acid followed by taurolithodeoxycholic acid predominated the bile acids in colostrum from the sows fed SBP and LNC (Figure 1a).

**Table 2.** Concentration (µg/mL) of bile acids in colostrum (collected within 10 hours after farrowing) from the sows fed diets containing high-fermentable sugar beet pulp or low-fermentable lignocellulose fibers during gestation and lactation

	SBP	LNC	*p* *
CDCA	0.87 ± 0.06	0.79 ± 0.06	0.386
GHCA	0.39 ± 0.05	0.27 ± 0.04	0.100
HCA	0.31 ± 0.05	0.37 ± 0.06	0.415
TCDCA	0.20 ± 0.04	0.21 ± 0.04	0.805
THCA	0.08 ± 0.01	0.16 ± 0.03	0.039
TDCA	0.04 ± 0.01	0.03 ± 0.002	0.095
THDCA	1.57 ± 0.21	1.39 ± 0.24	0.597
LCA	0.44 ± 0.10	0.10 ± 0.02	0.005
GHDCA	0.14 ± 0.02	0.28 ± 0.04	0.002
HDCA	1.60 ± 0.35	0.89 ± 0.14	0.080
OLCA	0.31 ± 0.07	0.08 ± 0.02	0.003
TLCA	0.12 ± 0.05	0.03 ± 0.004	0.122
Total BA	6.49 ± 0.48	6.00 ± 0.83	0.639
Primary BA	1.76 ± 0.185	1.94 ± 0.22	0.560
Secondary BA	4.73 ± 0.41	4.06 ± 0.65	0.421

SBP, sugar beet pulp; LNC, lignocellulose; LCA, lithocholic acid; DCA, deoxycholic acid; CDCA, chenodeoxycholic acid; HDCA, hyodeoxycholic acid; UDCA, ursodeoxycholic acid; CA, cholic acid; HCA, hyocholic acid; GCA, glycocholic acid; TDCA, taurodeoxycholic acid; TCA, taurocholic acid; TCDCA, taurochenodeoxycholic acid; THDCA, taurolithodeoxycholic acid; TUDCA, tauroursodeoxycholic acid; THCA, 3α,7α,12α-trihydroxycholestanoic acid; GCDCA, glycochenodeoxycholic acid; GHDCA, glycohyodeoxycholic acid; GHCA, glycohyocholic acid; OLCA, oxalolithocholic acid; TLCA, taurolithocholic acid.

* Unpaired Student t-test (significance at *p* ≤ 0.05).

### BA composition in the gallbladder bile of piglets

In the gallbladder, the concentration of taurochenodeoxycholic acid was significantly higher in the bile from LNC *vs*. SBP piglets (*p* = 0.035), while cholic acid and taurodeoxycholic acid were elevated in piglets from the SBP *vs*. LNC group (*p* = 0.018 and *p* = 0.001, respectively). The concentration of total, primary and secondary BA in the gallbladder bile of 4-6-day old animals was not different between the SBP and LNC groups (Table 3). Glycohyocholic acid, 3α,7α,12α-trihydroxycholestanoic acid, taurochenodeoxycholic acid, glycochenodeoxycholic acid and taurolithodeoxycholic acid were most abundant BA in the gallbladder bile in piglets from both feeding groups (Figure 1b).

**Table 3.** Concentration (mg/mL) of bile acids in the gallbladder bile from 4-6-day-old piglets whose dams were fed diets containing high-fermentable sugar beet pulp or low-fermentable lignocellulose fibers during gestation and lactation

	SBP	LNC	*p* *
CDCA	1291 ± 16.49	1268 ± 9.192	0.253
GHCA	8569 ± 1459	8232 ± 1035	0.856
HCA	767.4 ± 228.2	486.1 ± 206.7	0.375
TCDCA	5794 ± 997.2	10993 ± 2047	0.035
THCA	7954 ± 1073	12389 ± 2444	0.114
CA	96.7 ± 21.58	36.1 ± 8.512	0.018
TCA	359.2 ± 67.95	398.2 ± 75.18	0.705
TDCA	121 ± 25.09	13.6 ± 2.64	0.001
THDCA	4735 ± 1111	4652 ± 1034	0.957
TUDCA	134.2 ± 30.59	109.3 ± 27.6	0.557
GCA	400 ± 102.9	253.6 ± 39.84	0.220
GCDCA	5434 ± 1096	6976 ± 1533	0.424
GHDCA	2897 ± 556.8	2225 ± 387.3	0.336
Total BA	40014 ± 3265	54767 ± 9191	0.148
Primary BA	24395 ± 2154	32533 ± 4330	0.110
Secondary BA	51951 ± 4523	60317 ± 6996	0.328

SBP, sugar beet pulp; LNC, lignocellulose; LCA, lithocholic acid; DCA, deoxycholic acid; CDCA, chenodeoxycholic acid; HDCA, hyodeoxycholic acid; UDCA, ursodeoxycholic acid; CA, cholic acid; HCA, hyocholic acid; GCA, glycocholic acid; TDCA, taurodeoxycholic acid; TCA, taurocholic acid; TCDCA, taurochenodeoxycholic acid; THDCA, taurolithodeoxycholic acid; TUDCA, tauroursodeoxycholic acid; THCA, 3α,7α,12α-trihydroxycholestanoic acid; GCDCA, glycochenodeoxycholic acid; GHDCA, glycohyodeoxycholic acid; GHCA, glycohyocholic acid; OLCA, oxalolithocholic acid; TLCA, taurolithocholic acid.

* Unpaired Student t-test (significance at *p* ≤ 0.05).

### BA composition in the ileal digesta of piglets

In the ileal digesta, among the analysed BA, lithocholic acid was significantly higher in the piglets from the SBP *vs*. LNC group (*p* = 0.015). The concentration of total, primary and secondary BA in the ileal digesta was not different between the SBP and LNC groups (Table 4). Hyocholic acid followed by 3α,7α,12α-trihydroxycholestanoic acid predominated the bile composition in the ileal digesta from piglets from both feeding groups, while taurochenodeoxycholic acid was highly abundant in the bile from piglets from LNC group (Figure 1c).

**Table 4.** Concentration (mg/mL) of bile acids in the ileal digesta from 4-6-day-old piglets whose dams were fed diets containing high-fermentable sugar beet pulp or low-fermentable lignocellulose fibers during gestation and lactation

	SBP	LNC	*p* *
CDCA	757.7 ± 272.2	463.4 ± 192.3	0.402
GHCA	664 ± 377.3	828.2 ± 381.8	0.764
HCA	4139 ± 1093	4138 ± 1340	1.000
TCDCA	234.5 ± 111.1	2153 ± 1018	0.115
THCA	1874 ± 819.1	3568 ± 1208	0.273
LCA	8.5 ± 2.69	1.33 ± 0.5528	0.015
DCA	23.5 ± 6.78	16.8 ± 7.93	0.529
UDCA	22.9 ± 9.22	3.63 ± 1.43	0.084
CA	63.4 ± 25.7	80.6 ± 29.2	0.669
HDCA	381.4 ± 126.9	436.2 ± 185.2	0.815
TCA	103.4 ± 52.3	336.7 ± 132.3	0.134
TDCA	6.22 ± 3.78	5.3 ± 4.121	0.872
THDCA	227 ± 106.3	472 ± 245.1	0.390
TUDCA	21 ± 7.74	54.6 ± 29.1	0.279
GCA	23.8 ± 10.9	34.7 ± 17.3	0.602
GCDCA	138.4 ± 86.2	136.3 ± 101.5	0.987
GHDCA	37.6 ± 17.0	59.7 ± 34.9	0.577
Total BA	8871 ± 2674	16215 ± 5610	0.271
Primary BA	7344 ± 1995	11048 ± 3354	0.355
Secondary BA	9068 ± 2513	14001 ± 4331	0.338

SBP, sugar beet pulp; LNC, lignocellulose; LCA, lithocholic acid; DCA, deoxycholic acid; CDCA, chenodeoxycholic acid; HDCA, hyodeoxycholic acid; UDCA, ursodeoxycholic acid; CA, cholic acid; HCA, hyocholic acid; GCA, glycocholic acid; TDCA, taurodeoxycholic acid; TCA, taurocholic acid; TCDCA, taurochenodeoxycholic acid; THDCA, taurolithodeoxycholic acid; TUDCA, tauroursodeoxycholic acid; THCA, 3α,7α,12α-trihydroxycholestanoic acid; GCDCA, glycochenodeoxycholic acid; GHDCA, glycohyodeoxycholic acid; GHCA, glycohyocholic acid; OLCA, oxalolithocholic acid; TLCA, taurolithocholic acid.

* Unpaired Student t-test (significance at *p* ≤ 0.05).

### BA composition in the colonic digesta of piglets

In the colonic digesta, lithocholic acid and ursodeoxycholic acid were significantly higher in piglets from the SBP *vs*. LNC group (*p* = 0.001 and *p* = 0.007, respectively). The concentration of total, primary and secondary BA in the colonic digesta was not different between the SBP and LNC groups (Table 5). There was a trend for a higher concentration of secondary BA in piglets from SBP compared to LNC group (*p* = 0.007). Hyocholic acid, hyodeoxycholic acid and chenodeoxycholic acid were the most abundant BA in the colon digesta from piglets from SBP group, while hyocholic acid was the most abundant BA in the colon digesta from the piglets from LNC group (Figure 1d).

**Table 5.** Concentration (mg/mL) of bile acids in the colonic digesta from 4-6-day-old piglets whose dams were fed diets containing high-fermentable sugar beet pulp or low-fermentable lignocellulose fibers during gestation and lactation

	SBP	LNC	*p* *
CDCA	78.9 ± 26.8	74.8 ± 36.5	0.929
HCA	272.7 ± 86.5	766.4 ± 289.6	0.128
LCA	48.1 ± 8.62	10.5 ± 3.44	0.001
DCA	9.75 ± 2.37	7.71 ± 1.87	0.520
HDCA	125 ± 34.8	51.2 ± 14.0	0.107
UDCA	30.1 ± 5.83	10.6 ± 1.85	0.007
CA	5.14 ± 1.93	17.1 ± 6.80	0.115
Total BA	813.1 ± 286.8	808.1 ± 289.9	0.990
Primary BA	338.9 ± 108.1	822.3 ± 346.9	0.232
Secondary BA	213 ± 47.8	98.6 ± 33.2	0.070

SBP, sugar beet pulp; LNC, lignocellulose; LCA, lithocholic acid; DCA, deoxycholic acid; CDCA, chenodeoxycholic acid; HDCA, hyodeoxycholic acid; UDCA, ursodeoxycholic acid; CA, cholic acid; HCA, hyocholic acid; GCA, glycocholic acid; TDCA, taurodeoxycholic acid; TCA, taurocholic acid; TCDCA, taurochenodeoxycholic acid; THDCA, taurolithodeoxycholic acid; TUDCA, tauroursodeoxycholic acid; THCA, 3α,7α,12α-trihydroxycholestanoic acid; GCDCA, glycochenodeoxycholic acid; GHDCA, glycohyodeoxycholic acid; GHCA, glycohyocholic acid; OLCA, oxalolithocholic acid; TLCA, taurolithocholic acid.

* Unpaired Student t-test (significance at *p* ≤ 0.05).

## Discussion

Dietary fibre influences the intestinal microbiota composition and activity in pigs.^(1)^ Moreover, dietary fibre and its metabolites can have an impact on the intestinal physiology, immune system and may influence the BA homeostasis and host health.^(1,8)^ The current study assessed whether fibre-enriched diets (either sugar beet pulp or lignocellulose) fed to sows during gestation and lactation have any distinct impact on the BA composition in sow colostrum and in gallbladder bile, and jejunal and colonic digesta collected from their 4-6-day-old offspring.

Here, we were capable of quantifying 12 BA species in colostrum. A more detailed analysis revealed that specific primary and secondary BA showed differences in colostrum from the two dietary groups. Specifically, secondary BA such as lithocholic and oxolithocholic acids were significantly increased in colostrum from sows who consumed diets enriched in sugar beet pulp, while the opposite fibre effect was observed for the primary 3α,7α,12α-trihydroxycholestanoic and secondary glycohyodeoxycholic BA. Therefore, high-fermentable fibre diets promoted elevated concentrations of secondary BA in colostrum, which might have resulted from the increased microbial bile acid transformations in the gut of the sows. Sow colostrum is known to be rich in nutrients and immunoglobulins.^(14)^ The specific immunoglobulins present in colostrum play a crucial role in shielding piglets from environmental antigens and enteric infections.^(21)^ We have previously identified biogenic amines and anti-*C. difficile*-toxin-IgG-antibodies in sow colostrum.^(13,25)^ However, reports on the BA in colostrum are very scarce. Recently, the profiling of metabolites in sow colostrum from three breeds of pigs confirmed the existence of breed-specific bile acids signatures.^(16)^ In addition, two BA were recently quantified in colostrum from sows of different parity, but no differences were observed.^(15)^ In our study, we did not observe any significant differences in bile acid composition between gilts and sows. To our knowledge, however, our study is the first to quantify a wide range of BA in sow colostrum, thereby providing a more detailed BA profile of porcine colostrum. Although present at two- to four-fold lower concentration than in the piglet’s gut, their contribution to the biological actions of the colostrum and potential implications in early life of the suckling offspring warrants further investigation. BA have been detected in colostrum of healthy women (0.7 ± 0.2 µM of total BA) while women with intrahepatic cholestasis of pregnancy exhibited elevated levels of total BA (23.3 ± 14.8 µM).^(26)^ It is possible that the presence of BA in colostrum in a healthy state has relevant biological functions. In fact, BA that were altered by the feeding of highly-fermentable fibre in this study are known to regulate intestinal barrier function, cell proliferation and immune function.^(3)^ In light of their pleiotropic actions, therefore, BA in colostrum could certainly have implications for the colostrum nutritional quality and potential health benefits in new-born piglets, such as promotion of intestinal barrier function through FXR activation.^(3)^

Another aspect that remains poorly understood is the origin of BA in colostrum. BA have been detected in healthy murine brain (ca. 25 ng/g), which may suggest their signalling role on the central nervous system.^(27)^ A possible source of colostral BA could be through the systemic blood. However, the presence of BA in systemic blood (15–87 µM) has been related to serious illnesses such as hepatic failure in humans.^(28)^ Nevertheless, it is possible that small amounts of BA, without a harming effect, enter the systemic blood circulation after absorption in the gut from where they reach the mammary glands. Whether the type of fibre in the diet, either high- or low-fermentable, would have any impact on the BA transport to colostrum in sows, needs further investigation.

We have observed a few significant differences in the concentrations of primary and secondary BA from the gallbladder bile from both experimental groups of piglets. It is known that the fermentation of dietary fibres produces SCFA, which can affect BA synthesis in the liver. Particularly butyrate, has been shown to influence the expression of genes involved in BA metabolism.^(29)^ Therefore, a link could exist between fibre-induced changes and the efficiency of fat digestion through the increased detergent properties of conjugated BA in the gastrointestinal tract. It is known that fat digestion begins in the duodenum by the secretion of bile and continues in the jejunum and ileum, where emulsified fats can be absorbed by the intestinal epithelium. Indeed, conjugated BA, such as the primary taurochenodeoxycholic acid and the secondary taurodeoxycholic acid, which showed group differences, have increased detergent properties and are therefore far more efficient at fat emulsification and digestion than unconjugated BA.^(30)^ Interestingly, high concentrations of secondary bile acids in piglet bile may result from early microbial biotransformation in the intestine combined with enterohepatic recirculation, through which secondary metabolites are reabsorbed, conjugated in the liver, and secreted back into the bile.^(31)^

We have demonstrated that piglets from the sugar beet pulp group had either significantly or numerically higher concentration of major secondary BA such as lithocholic acid and ursodeoxycholic acid, in the ileal digesta, as compared to piglets from the lignocellulose group. Pigs are recognised for their tendency towards coprophagy, and nursing piglets maintain constant exposure to the sow’s faeces after birth. This close contact may potentially impact the gut microecology of the offspring, influenced by that of the sow.^(32)^ Sugar beet pulp leads to a higher extent of microbial fermentation due to the presence of fermentable pectin and other carbohydrates. A modified metabolic activity of the piglet small intestinal microbiota, especially towards the end of the small intestine, might have contributed to BA deconjugation and higher lithocholic and ursodeoxycholic acids in this group of piglets. It is known that especially lactobacilli from the small intestine contain BA deconjugating enzymes,^(33)^ which may in part explain the higher concentration of these BA in the piglets from the sugar beet pulp group. Additionally, BA are reabsorbed in the ileum, and their concentration can be influenced by the types of fibres present in the diet. Dietary fibre, especially insoluble fibre such as lignocellulose, can influence gut motility.^(34)^ Changes in the gut motility can affect the residence time of BA and bacteria in the small intestine, influencing their absorption and deconjugation, which could in part explain the observed differences in BA from the two dietary fibre groups. In addition, the interaction between BA and dietary fibres can affect the solubility and absorption of BA in the small intestine.^(35)^

A small portion of microbially-produced (secondary) BA escapes reabsorption in the ileum and is transformed by colonic microbiota.^(2)^ Some of these secondary deconjugated and dexydroxylated BA can effectively influence microbial populations by either inhibiting or stimulating bacterial growth and activity. For instance, taurocholic, glycocholic and cholic acids are produced by the liver and act as potential germinant of *C. difficile* spores, whereas chenodeoxycholic acid inhibits germination and the secondary species deoxycholic and lithocholic acids inhibit growth and toxin synthesis by vegetative cells of *C. difficile*.^(36)^ Reports also show that, deoxycholic and chenodeoxycholic acids display toxicity against *Bifidobacterium breve*, *Blautia coccoides* and *Bacteroides thetaiotaomicron*.^(37)^

Higher concentrations of some secondary BA such as deoxycholic acid, lithocholic acid, taurodeoxycholic acid, or the primary BA such as chenodeoxycholic acid, may alter epithelial barrier function, cell signalling and immune response.^(3,38)^ Here, we showed that the total secondary BA including their species such as lithocholic and ursodeoxycholic acids were increased in the piglets whose dams were fed sugar beet pulp, as compared to lignocellulose. Higher concentrations of secondary BA may reflect increased microbial activity due to high-fermentable fibres, compared to low-fermentable fibres. Interestingly, we have previously reported that the expression of zonula occludens 1 (ZO-1) and transforming growth factor β (TGF-β) genes in the colonic tissue were upregulated in the piglets from the sows fed sugar beet pulp as compared to lignocellulose.^(13)^ Indeed, micromolar concentrations of cholic, deoxycholic and chenodeoxycholic acids but not ursodeoxycholic acid decreased transepithelial electrical resistance and increased paracellular permeability in a Caco-2 cell model.^(39)^ In addition, elevated cholic acid in the colon has been associated with inflammation or disease processes in the gut,^(40)^ which may enhance the expression and activation of TGF-β, contributing to tissue damage and remodelling.^(39)^

BA, depending on their concentration and origin (primary or secondary), have been reported to either stimulate or inhibit the intestinal epithelial cell proliferation. Specifically, higher concentrations of certain secondary BA have inhibitory or cytotoxic activity while low concentrations can be beneficial to the epithelial cells.^(41)^ Dietary fibre influences colonic secondary lithocholic BA levels, as seen in previous studies. For instance, the inclusion of soluble flaxseed meal in feed, compared to insoluble oat hulls, has been shown to increase faecal lithocholic acid concentration in growing pigs (ca. 25 kg body weight) (3.97 *vs*. 2.26 mg/g).^(42)^ Despite this, knowledge regarding the effect of lithocholic acid on porcine intestinal cells remains limited. Nevertheless, in cancer research, the impact of lithocholic acid on the inhibition of colonic cell proliferation is widely studied.^(4)^ In our previous observations, significant increases in colonic proliferating cell nuclear antigen gene expression (PCNA) were noted in piglets from the sugar beet pulp group, which coincided with higher lithocholic acid concentrations in this group.^(13)^

The body weight of piglets or sow feed intake were unlikely to affect BA levels in colostrum and intestinal digesta, respectively, as these characteristics were controlled and remained similar across both feeding groups. Besides, reports on the impact of dietary fibre on animal performance and relation to BA levels in colostrum or intestinal digesta are scarce.^(6)^ This feeding experiment would benefit from the microbiota composition and metabolomics analysis in intestinal digesta of the study sows and piglets. Such data could offer valuable insights into the microbial activity’s potential to metabolise BA. Additionally, in future BA analysis of sow milk should be performed, to allow potential associations with BA levels in piglet digesta. However, such analysis goes beyond the scope of the current study aims.

Insights gained from the current study may also contribute to understanding how maternal diet influences BA profiles in colostrum and infant gut. These findings could support dietary strategies to enhance maternal-infant health in human medicine. The research may also provide valuable implications for preventing BA-related diseases and optimising nutrient absorption through targeted dietary interventions. The role of BA in colostrum in healthy mothers should be further investigated and pigs can be considered as valuable model species.

In summary, incorporating specific dietary fibres into the diet of sows could modify the composition of certain BA in their colostrum. BA in colostrum may affect the BA balance in piglets. Additional studies are needed to fully understand how fibre-rich diets influence the presence of BA in colostrum, the digestive system of new-born piglets, and the broader aspect of piglet health.
